# Relationship between Anemia and Readmission among Older Patients in Rural Community Hospitals: A Retrospective Cohort Study

**DOI:** 10.3390/jcm13020539

**Published:** 2024-01-18

**Authors:** Shiho Amano, Ryuichi Ohta, Chiaki Sano

**Affiliations:** 1 Community Care, Unnan City Hospital, Daito-cho Iida, Unnan 699-1221, Japan; ryuichiohta0120@gmail.com; 2 Department of Community Medicine Management, Faculty of Medicine, Shimane University, Enya-cho, Izumo 693-8501, Japan; sanochi@med.shimane-u.ac.jp

**Keywords:** anemia, readmission, older patients, Japan, rural community hospital

## Abstract

Readmission rates among older adults are a growing concern, and the association of readmission with anemia and the potential benefits of a systematic assessment and intervention remain unclear. This study investigated the association between anemia and readmission within 28 and 90 days in an older population. Data from 1280 patients admitted to the Department of General Medicine of Unnan City Hospital between April 2020 and December 2021 were retrospectively analyzed. Variables such as anemia status, Charlson comorbidity index (CCI) score, Functional Independence Measure (FIM) score, and dependent status were evaluated. Multivariate logistic regression was used to determine the associations between 28-day and 90-day readmissions. The average age was 84.9 years, and the prevalence of anemia was 36.4%. The readmission rates within 28 and 90 days were 10.4% and 19.1%, respectively. Anemia was significantly associated with readmission in both periods (28-day adjusted odds ratio, 2.28; 90-day adjusted odds ratio, 1.65). CCI score, FIM score, and dependent status were also identified as significant factors. Anemia is significantly associated with short- and medium-term readmissions in older patients. Addressing anemia, along with other identified factors, may help reduce readmission rates.

## 1. Introduction

Anemia is a prevalent condition in older adults, and its presence correlates with increased mortality and hospitalization rates. The effective management of anemia in older adults is paramount and requires rigorous assessment. According to the World Health Organization, anemia is characterized by hemoglobin (Hb) levels of <13 g/dL in males and <12 g/dL in females [1]. Among individuals aged > 65 years, the prevalence of anemia is 17%; however, it increases to 40% in hospitalized patients and 12% in community settings [2]. An increase in the prevalence of anemia was observed with advancing age [3]. The specific definition of anemia in older adults remains ambiguous because the lower Hb threshold in healthy older individuals is not markedly different from that in their younger counterparts [4]. Numerous studies have linked anemia to increased mortality rates, increased hospitalization, dementia, falls, diminished physical function, and decreased quality of life (QOL) in older adults [3]. A cohort study involving 17,030 individuals aged 66 and above demonstrated an inverse J-shaped correlation between Hb levels and all-cause mortality and hospitalization due to cardiovascular events [5]. Minimal mortality risk was observed at Hb ranges of 13–15 g/dL for females and 14–17 g/dL for males, regardless of chronic kidney disease (CKD) status [3,5]. Given that the definition of anemia in older adults aligns with that in younger populations, its management should be approached in a similar manner across different age groups.

In a previous study, we examined anemia recognition among older adults in a rural community hospital [6]. The findings indicated that only 40.4% of older patients in this setting were identified as having anemia. Multivariate logistic regression analysis revealed that age was the only significant predictor of anemia recognition. This association suggests potential ageism in anemia recognition among the older adult population. Ageism, which refers to stereotypes, prejudices, and discrimination based on age, has been consistently linked to adverse physical and psychological health outcomes [7,8,9]. Although older adults deserve comprehensive medical care, age-based limitations may persist. While there is a clear association between anemia and increased mortality and hospitalization in older adults, the potential benefits of systematic assessment and intervention remain unclear.

Although our previous research highlighted a suboptimal rate of anemia recognition, the influence of anemia on outcomes, such as readmission rates, remains ambiguous. Moreover, the impact of anemia diagnosis and treatment on outcomes, such as readmission rates, is yet to be determined. Frequent hospital readmissions in older patients have economic and prognostic ramifications [10,11]. Some of these readmissions may be avoidable, and the economic repercussions of preventable readmissions have been documented [12]. Notably, hospital readmissions in older individuals correlate with increased 1-year mortality rates. However, it remains uncertain whether mitigating readmissions directly enhances survival [11]. This study aimed to ascertain whether anemia in older adults is correlated with readmission rates and the interval between discharge and subsequent readmission.

## 2. Materials and Methods

### 2.1. Setting

Unnan City Hospital, located in the southeastern part of Shimane Prefecture, Japan, is a rural community hospital and the only public hospital in the city. The hospital team consisted of 27 physicians, 197 nurses, seven pharmacists, 15 clinical technicians, 37 therapists (22 physical therapists, 12 occupational therapists, three speech therapists), four nutritionists, and 34 clerks. No other medical institution is equipped with a recovery or rehabilitation unit in its vicinity. In 2023, the population of Unnan City was 35,686 (17,199 males and 18,487 females), with 40.27% aged > 65 years [13].

### 2.2. Participants

The study focused on a geriatric cohort, encompassing patients aged 65 years and above. This deliberate choice was made to gain insights into the health outcomes and medical needs of older adults, a demographic that often faces unique medical challenges. Our research included only patients admitted to the Department of General Medicine at Unnan City Hospital. This department was selected due to its broad spectrum of patient care, offering a rich and diverse patient profile for assessing general health conditions prevalent in older adults. We ensured the integrity of our research by including only patients with comprehensive and complete electronic medical records. In our pursuit of focused and relevant data, we excluded patients admitted to departments other than General Medicine, which was vital to maintain the specificity and relevance of our study to general health conditions; individuals who were admitted for routine medical checkups; and patients with any gaps or incompleteness in their electronic medical records.

### 2.3. Data Collection

Data were retrospectively collected from the electronic medical records of the hospital. This study considered readmissions within 28 and 90 days as the dependent variables. Though the lower limit for normal Hb in older patients remains undefined, one Swedish study involving healthy participants aged 70–88 determined a lower limit of 11.5 g/dL for those aged 80–82 years [14]. In the older Japanese population, a threshold of 11.0 g/dL is commonly used [15]. Given that the average age of our participants was 84.9 years, anemia was defined as an Hb level less than 11.0 g/dL. Based on previous studies, risk factors for readmission were identified and assessed as independent variables [11]. Data on these variables, including age, sex, albumin level, BMI, dependent conditions, Charlson comorbidity index (CCI) [16], admission and discharge facilities, Functional Independence Measure (FIM) score on admission [17], number of medications, Hb level, admission duration, readmission occurrence, time to readmission, and readmission within 28 and 90 days, were derived from the electronic medical records. Laboratory metrics, such as albumin and Hb levels, were extracted from tests conducted on the day of admission. FIM values were recorded by a nurse through interviews at the time of admission. Dependent status was defined as a level of care needs that corresponds to the use of long-term care insurance, which is one of the Japanese social insurance systems, and it is graded from 1 to 5 (with 5 indicating that the individual requires the most care).

### 2.4. Statistical Analysis

For continuous variables, data normality was confirmed before statistical testing. The *t*-test and Mann–Whitney U test were applied to parametric and nonparametric data, respectively. Fisher’s exact test was used for nominal variables. Certain continuous variables were dichotomized for binary analysis: CCI (either > 5 or <5) [16] and dependent condition (either dependence ≥ 1 or 0) [18]. To explore the correlation between readmission occurrence and other influential factors, multivariate logistic regression analysis was performed. Only variables that were correlated with anemia in the univariate regression analysis were considered in the multivariate logistic model. All statistical analyses were conducted using Easy R (version 1.54; R Foundation for Statistical Computing, Vienna, Austria), with the significance level set at *p* < 0.05 [19].

### 2.5. Ethical Considerations

Patient information anonymity and confidentiality are of utmost importance. Both the patients and their family members were briefed on the use of their medical records for this study, and informed consent was obtained. A summary of the research, excluding personal patient details, is accessible on the website of the hospital. Contact details for the hospital representative were available, ensuring that any questions regarding the study could be addressed promptly. All the study procedures were performed in accordance with the Declaration of Helsinki and its subsequent amendments. This study was approved by the Clinical Ethics Committee of Unnan City Hospital (approval number: 20210005; date: 18 May 2021).

## 3. Results

### 3.1. Demographics of Participants

**Figure 1:**
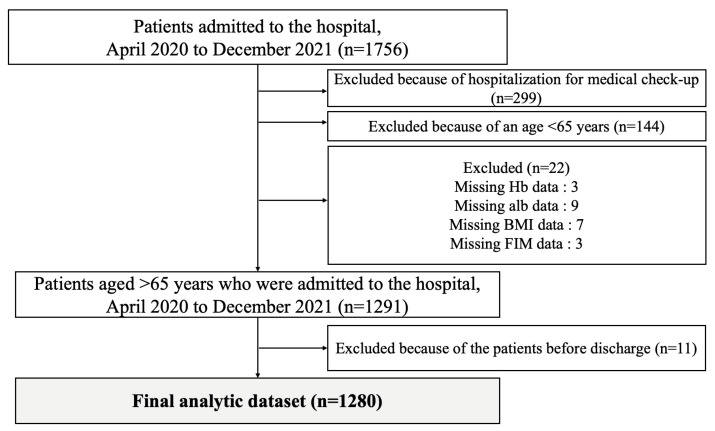
Demographics of participants.

Figure 1 shows a flowchart of the study population selection process. From April 2020 to December 2021, 1756 patients were admitted to the Department of General Medicine of Unnan City Hospital. A total of 299, 144, and 22 patients were excluded because of hospitalization for medical checkups, age < 65 years, or incomplete data (no Hb data, 3; no albumin data, 9; no BMI data, 7; and no FIM data, 3), respectively. Eleven patients were excluded because they were still hospitalized at the time of the study. In total, 1280 patients were included in this study.

### 3.2. Characteristics of the Study Participants

Table 1 shows the characteristics of the study participants. The mean patient age was 84.9 years (standard deviation [SD] = 8.4). The prevalence of anemia was 36.4%. The prevalence of readmission was 24.9%, and the average readmission duration was 232.4 days (SD = 190.2). The prevalences of readmission within 28 and 90 days were 10.4% and 19.1%, respectively. The average length of hospital stay was 25 days (SD = 28.0). The proportion of patients who were admitted from a nursing home was 23.0% and the proportion discharged to a nursing home was 29.1% (Table 1).

### 3.3. Association between Anemia and Readmission

Table 2 shows the univariate regression analysis of patient characteristics for anemia. Age, sex, BMI, Alb level, dependent condition, CCI, duration of admission, presence of readmission, and number of medications differed significantly between the anemia and no-anemia groups. The FIM score was not associated with anemia (Table 2). Multivariate logistic regression was performed on the factors associated with readmission within 28 and 90 days. Age, sex, BMI, anemia, dependent conditions, CCI, and FIM were included as factors. Readmission within 28 days was significantly associated with anemia (adjusted odds ratio = 2.28, 95% confidence interval [CI]: 1.56–3.33, *p* < 0.001), CCI (adjusted odds ratio = 2.03, 95% CI: 1.23–3.36, *p* = 0.006), and FIM (adjusted odds ratio = 1.01, 95% CI: 1.00–1.01, *p* = 0.032) (Table 3). Readmission within 90 days was significantly associated with anemia (adjusted odds ratio = 1.65, 95% CI: 1.21–2.24, *p* = 0.002), dependent status (adjusted odds ratio = 1.55, 95% CI: 1.03–2.33, *p* = 0.037), CCI (adjusted odds ratio = 2.19, 95% CI: 1.48–3.24, *p* < 0.001), and FIM (adjusted odds ratio = 1.01, 95% CI: 1.01–1.01, *p* < 0.001) (Table 4).

## 4. Discussion

Our findings revealed a potential association between anemia and readmission within 28 and 90 days. With an average participant age of 84.9 years and an anemia prevalence of 36.4%, our findings align with those of previous studies concerning the prevalence of anemia [2,6]. Given that our hospital is the sole public facility in the city and predominantly caters to the older population, we observed a higher trend in readmission rates.

Notably, while early readmission (within 28 days) appeared to be influenced by factors such as anemia, CCI index, and FIM score, 90-day readmission was associated with anemia, dependent status, FIM score, and CCI index. In contrast, age, sex, and BMI were not significantly associated with readmission. We believe that all these factors are very important. CCI can be improved with appropriate intervention. Improving activities of daily living (ADL) in older adults is relatively difficult. However, previous studies have shown that anemia in older adults is more than half of the time unrecognized, and such adults may not be receiving the necessary intervention and treatment due to ageism [6]. Therefore, we selected anemia to demonstrate the potential for improvement in rehospitalization rates if anemia is appropriately identified and assessed.

The association between anemia and readmission, as demonstrated in previous studies, highlights the propensity of patients with anemia to experience complications such as lower respiratory tract infections, chronic obstructive pulmonary disease (COPD), and postoperative conditions [20,21,22]. Furthermore, the severity of anemia is directly correlated with increased readmission risk [23]. Our study not only echoes this sentiment but also underscores the potential association between comorbidities, ADL, and readmission rates. Interestingly, while addressing comorbidities and ADL might pose challenges, detecting and treating anemia, especially in the older population, seems more straightforward. However, the condition is often undetectable in older adults [6]. Whether addressing anemia can result in reduced readmission remains an open question and requires further research.

The staggering readmission rate of 24.9% in our study, with 10.4% and 19.1% readmissions within 28 and 90 days, respectively, diverged significantly from the national average in Japan. According to a 2021 report by the Japan Hospital Organization, the 30-day readmission rate ranged from 0.0–6.5% [24]. Although our study did not delve into the specific reasons for these readmissions, previous research indicated links between early readmissions and conditions such as heart failure, stroke, and COPD [11]. Other determinants of early readmission include high CCI score, prolonged hospitalization, inadequate patient education upon discharge, and medical emergency team (MET) calls [25,26,27]. Interestingly, another Australian retrospective study discovered that factors such as malnutrition, weekday discharge, and Indigenous status increased the risk of early readmission [26].

Age as a determinant of readmission remains a topic of debate. Existing literature presents varied conclusions—some suggest improved outcomes with age, others indicate the opposite, and some show no discernible relationship [25,26,27]. Our findings, based on the multivariate analysis, did not identify age as a significant factor for readmission. Previous studies have also highlighted marital status, particularly single or divorced, as a determinant of readmission risk [26,28]. The existing literature remains divided regarding functional impairment and readmission [29,30,31]. It is worth noting the uniqueness of the demographics of our hospital; being a rural institution with an older average patient age of 84.9 years, a propensity towards malnutrition, and multiple comorbidities led to a higher readmission rate compared to national statistics.

In our previous study, anemia was observed in only 40.4% of older inpatients [6]. Age was the only factor that showed a significant association with anemia recognition. Therefore, appropriate medical care should be provided to older patients. However, the results of this study indicate that we may be practicing ageism, which inappropriately limits medical care based on age. Early rehospitalization and mortality are associated with anemia; however, whether the assessment and treatment of anemia can improve outcomes needs to be clarified [3].

There are several limitations in our study. External validity may have been compromised in this single-center study. However, our demographics are reflective of the impending global demographic shift towards an aging population, which not only Japan but also many other countries worldwide will soon grapple with. These data cover the period from April 2020 to December 2021, the time frame within which the coronavirus disease 2019 pandemic occurred. Therefore, the external validity of the study may have been reduced because the consultation behavior may have been different than usual. However, we assessed the consultation behavior of older adults during the pandemic in this study. The retrospective nature of this study brings its own set of challenges, including unknown variables related to the social aspects of anemia and readmission. Moreover, this study did not evaluate the correlation between specific diseases and readmission rates. Anemia can be caused by a variety of factors, and the results may differ depending on the underlying disease and degree of anemia. However, this study did not examine these factors, and further research is needed.

## 5. Conclusions

Our research highlights the potential association between anemia and readmission rates, both within the short- (28 days) and medium-term (90 days) time frames. Factors such as anemia, CCI score, and FIM score have emerged as determinants of short-term readmission. Addressing anemia may provide a pathway to reduce short-term readmissions regardless of disease severity, ADL status, or age.

## Figures and Tables

**Table 1:** Characteristics of the study participants.

Age (year), mean (SD)	84.9 (8.4)	FIM at admission, mean (SD)	74.0 (41.8)
Sex, male (%)	578 (45.2)	Number of medications, mean (SD)	6.3 (3.5)
Sex, female (%)	702 (54.8)	Hb (g/dL), mean (SD)	11.5 (2.2)
Albumin (g/dL), mean (SD)	3.4 (0.6)	Length of hospital stay (day), mean (SD)	25 (28.0)
BMI (kg/m^2^), mean (SD)	20.3 (3.7)	Readmission, *n* (%)	319 (24.9)
Dependent condition, *n* (%) (dependence ≥ 1)	620 (48.4)	Duration until readmission (day), mean (SD)	232.4 (190.2)
CCI score (≥5), *n* (%)	873 (68.2)	Readmission within 28 days (%)	134 (10.5)
Discharge to nursing home, *n* (%)	373 (29.1)	Readmission within 90 days (%)	245 (19.1)
Admission from nursing home, *n* (%)	295 (23.0)		

The data are presented as percentages and number of patients (*n*) or mean (standard deviation [SD]). BMI = body mass index, CCI = Charlson comorbidity index, FIM = Functional Independence Measure, Hb = hemoglobin.

**Table 2:** Association between anemia and other factors.

	Anemia		*p*-Value
	Negative (*n* = 814)	Positive (*n* = 466)	
Age (year), mean (SD)	83.6 (8.8)	87.2 (7.3)	<0.001
Sex, male (%)	388 (47.7)	190 (40.8)	0.019
Sex, female (%)	426 (52.3)	276 (59.2)	
Albumin (g/dL), mean (SD)	3.6 (0.6)	3.2 (0.6)	<0.001
BMI (kg/m^2^), mean (SD)	20.6 (3.7)	19.6 (3.6)	<0.001
Dependent condition, *n* (%) (dependence ≥ 1)	367 (45.1)	253 (54.3)	0.002
CCI score (≥5), *n* (%)	508 (62.4)	365 (78.3)	<0.001
Discharge to nursing home, *n* (%)	238 (29.2)	135 (29.0)	0.949
Admission from nursing home, *n* (%)	181 (22.2)	113 (24.2)	0.269
Duration of admission (day), mean (SD)	23.7 (25.4)	27.3 (31.8)	0.027
FIM at admission, mean (SD)	75.2 (42.2)	72.0 (40.9)	0.187
Readmission, *n* (%)	185 (22.7)	134 (28.8)	0.019
Number of medications, mean (SD)	6.0 (3.3)	6.9 (3.6)	<0.001

The data show the univariate regression analysis results between the two patient groups. BMI = body mass index, CCI = Charlson comorbidity index, FIM = Functional Independence Measure.

**Table 3:** Association between readmission within 28 days and influencing factors.

Factor	Odds Ratio	95% CI	*p*-Value
Age	1.00	0.98–1.03	0.867
Sex, male	1.41	0.97–2.06	0.075
Sex, female	0.71	0.49–1.03	
BMI	1.00	0.95–1.05	0.926
Anemia, positive	2.28	1.56–3.33	<0.001
Dependence ≥ 1	1.34	0.82–2.19	0.241
CCI ≥ 5	2.03	1.23–3.36	0.006
FIM	1.01	1.00–1.01	0.032

The data show multivariate logistic regression results of the factors associated with readmission within 28 days. BMI = body mass index, CCI = Charlson comorbidity index, FIM = Functional Independence Measure.

**Table 4:** Association between readmission within 90 days and influencing factors.

Factor	Odds Ratio	95% CI	*p*-Value
Age	1.01	0.98–1.03	0.578
Sex, male	1.31	0.96–1.78	0.090
Sex, female	0.77	0.56–1.04	
BMI	0.96	0.92–1.00	0.063
Anemia, positive	1.65	1.21–2.24	0.002
Dependence ≥ 1	1.55	1.03–2.33	0.037
CCI ≥ 5	2.19	1.48–3.24	<0.001
FIM	1.01	1.01–1.01	<0.001

The data show multivariate logistic regression results of the factors associated with readmission within 90 days. BMI = body mass index, CCI = Charlson comorbidity index, FIM = Functional Independence Measure.

## Data Availability

The datasets used and/or analyzed during the current study are available from the corresponding author on reasonable request.

## References

[1] World Health Organization (1968). Nutritional Anaemias. Report of a WHO Scientific Group.

[2] Lanier J.B., Park J.J., Callahan R.C. (2018). Anemia in older adults. Am. Fam. Physician.

[3] Stauder R., Thein S.L. (2014). Anemia in the elderly: Clinical implications and new therapeutic concepts. Haematologica.

[4] Beutler E., Waalen J. (2006). The definition of anemia: What is the lower limit of normal of the blood hemoglobin concentration?. Blood.

[5] Culleton B.F., Manns B.J., Zhang J., Tonelli M., Klarenbach S., Hemmelgarn B.R. (2006). Impact of anemia on hospitalization and mortality in older adults. Blood.

[6] Amano S., Ohta R., Sano C. (2021). Recognition of anemia in elderly people in a rural community hospital. Int. J. Environ. Res. Public Health.

[7] Sloane P.D., Gruber-Baldini A.L., Zimmerman S., Roth M., Watson L., Boustani M., Magaziner J., Hebel J.R. (2004). Medication undertreatment in assisted living settings. Arch. Intern. Med..

[8] Lang P.O., Hasso Y., Dramé M., Vogt-Ferrier N., Prudent M., Gold G., Michel J.P. (2010). Potentially inappropriate prescribing including under-use amongst older patients with cognitive or psychiatric co-morbidities. Age Ageing.

[9] Chang E.S., Kannoth S., Levy S., Wang S.Y., Lee J.E., Levy B.R. (2020). Global reach of ageism on older persons’ health: A systematic review. PLoS ONE.

[10] Jencks S.F., Williams M.V., Coleman E.A. (2009). Rehospitalizations among patients in the medicare fee-for-service program. N. Engl. J. Med..

[11] Lum H.D., Studenski S.A., Degenholtz H.B., Hardy S.E. (2012). Early hospital readmission is a predictor of one-year mortality in community-dwelling older medicare beneficiaries. J. Gen. Intern. Med..

[12] Medicare Payment Advisory Commission (2005). A path to bundled payment around a rehospitalization. Medicare Paym Advisory Comm Report to the Congress: Reforming the Delivery System.

[13] Unnan City Population and Number of Households in Unnan City (as of the End of November). https://www.city.unnan.shimane.jp/unnan/shiseijouhou/jouhoukoukai/toukei/jinkou.html.

[14] Nilsson-Ehle H., Jagenburg R., Landahl S., Svanborg A. (2000). Blood haemoglobin declines in the elderly: Implications for reference intervals from age 70 to 88. Eur. J. Haematol..

[15] Tsutsumi H., Ota M. (2006). Diagnosis and treatment of anemia. 4. Anemia of the aged. Nihon Naika Gakkai Zasshi.

[16] Charlson M.E., Pompei P., Ales K.L., MacKenzie C.R. (1987). A new method of classifying prognostic comorbidity in longitudinal studies: Development and validation. J. Chronic Dis..

[17] Linacre J.M., Heinemann A.W., Wright B.D., Granger C.V., Hamilton B.B. (1994). The structure and stability of the functional independence measure. Arch. Phys. Med. Rehabil..

[18] Ohta R., Maeki N., Maniwa S., Miyakoshi K. (2021). Predicting factors of elderly patients’ discharge to home after rehabilitation in rural japan: A retrospective cohort study. Rural Remote Health.

[19] Kanda Y. (2013). Investigation of the freely available easy-to-use software “EZR” for medical statistics. Bone Marrow Transplant.

[20] He Y., Chen Y., Cao K., Zheng H. (2022). Effect of anemia on readmission and death in octogenarian patients with lower respiratory tract infections: A retrospective cohort study. Int. J. Clin. Pract..

[21] Barba R., de Casasola G.G., Marco J., Emilio Losa J., Plaza S., Canora J., Zapatero A. (2012). Anemia in chronic obstructive pulmonary disease: A readmission prognosis factor. Curr. Med. Res. Opin..

[22] Segon Y.S., Dunbar S., Slawski B. (2021). Perioperative anemia: Clinical practice update. Hosp. Pract..

[23] Koch C.G., Li L., Sun Z., Hixson E.D., Tang A., Chagin K., Kattan M., Phillips S.C., Blackstone E.H., Henderson J.M. (2017). Magnitude of anemia at discharge increases 30-day hospital readmissions. J. Patient Saf..

[24] Japan Hospital Association https://www.hospital.or.jp/qipro/report/file/1674317922.pdf.

[25] Marcantonio E.R., McKean S., Goldfinger M., Kleefield S., Yurkofsky M., Brennan T.A. (1999). Factors associated with unplanned hospital readmission among patients 65 years of age and older in a Medicare managed care plan. Am. J. Med..

[26] Sharma Y., Miller M., Kaambwa B., Shahi R., Hakendorf P., Horwood C., Thompson C. (2018). Factors influencing early and late readmissions in Australian hospitalised patients and investigating role of admission nutrition status as a predictor of hospital readmissions: A cohort study. BMJ Open.

[27] Niu X.N., Wen H., Sun N., Zhao R., Wang T., Li Y. (2022). Exploring risk factors of short-term readmission in heart failure patients: A cohort study. Front. Endocrinol..

[28] Damiani G., Salvatori E., Silvestrini G., Ivanova I., Bojovic L., Iodice L., Ricciardi W. (2015). Influence of socioeconomic factors on hospital readmissions for heart failure and acute myocardial infarction in patients 65 years and older: Evidence from a systematic review. Clin. Interv. Aging.

[29] Tonkikh O., Shadmi E., Flaks-Manov N., Hoshen M., Balicer R.D., Zisberg A. (2016). Functional status before and during acute hospitalization and readmission risk identification. J. Hosp. Med..

[30] Hoyer E.H., Needham D.M., Atanelov L., Knox B., Friedman M., Brotman D.J. (2014). Association of impaired functional status at hospital discharge and subsequent rehospitalization. J. Hosp. Med..

[31] Aubert C.E., Folly A., Mancinetti M., Hayoz D., Donzé J.D. (2017). Performance-based functional impairment and readmission and death: A prospective study. BMJ Open.

